# Load magnitude affects patellar tendon mechanical properties but not collagen or collagen cross-linking after long-term strength training in older adults

**DOI:** 10.1186/s12877-019-1043-0

**Published:** 2019-01-31

**Authors:** Christian S. Eriksen, Rene B. Svensson, Anne T. Gylling, Christian Couppé, S. Peter Magnusson, Michael Kjaer

**Affiliations:** 1 Institute of Sports Medicine Copenhagen, Bispebjerg Hospital, Nielsine Nielsens Vej 11, building 8, 1st floor, DK-2400 Copenhagen, Denmark; 2 Center for Healthy Aging, Department of Health and Medical Sciences, University of Copenhagen, Blegdamsvej 3B, DK-2200 Copenhagen, N Denmark; 3 Department of Physical and Occupational Therapy, Bispebjerg Hospital, Nielsine Nielsens Vej 11, DK-2400 Copenhagen, Denmark

**Keywords:** Tendon biomechanics, Collagen cross-links., Strength training., Aging.

## Abstract

**Background:**

Regular loading of tendons may counteract the negative effects of aging. However, the influence of strength training loading magnitude on tendon mechanical properties and its relation to matrix collagen content and collagen cross-linking is sparsely described in older adults. The purpose of the present study was to compare the effects of moderate or high load resistance training on tendon matrix and its mechanical properties.

**Methods:**

Seventeen women and 19 men, age 62–70 years, were recruited and randomly allocated to 12 months of heavy load resistance training (HRT), moderate load resistance training (MRT) or control (CON). Pre- and post-intervention testing comprised isometric quadriceps strength test (IsoMVC), ultrasound based testing of in vivo patellar tendon (PT) mechanical properties, MRI-based measurement of PT cross-sectional area (CSA), PT biopsies for assessment of fibril morphology, collagen content, enzymatic cross-links, and tendon fluorescence as a measure of advanced glycation end-products (AGEs).

**Results:**

Thirty three participants completed the intervention and were included in the data analysis. IsoMVC increased more after HRT (+ 21%) than MRT (+ 8%) and CON (+ 7%) (*p* < 0.05). Tendon stiffness (p < 0.05) and Young’s modulus (*p* = 0.05) were also differently affected by training load with a reduction in CON and MRT but not in HRT. PT-CSA increased equally after both MRT and HRT. Collagen content, fibril morphology, enzymatic cross-links, and tendon fluorescence were unaffected by training.

**Conclusion:**

Despite equal improvements in tendon size after moderate and heavy load resistance training, only heavy. load training seemed to maintain tendon mechanical properties in old age. The effect of load magnitude on tendon biomechanics was unrelated to changes of major load bearing matrix components in the tendon core.

The study is a sub-study of the LISA study, which was registered at http://clinicaltrials.gov (NCT02123641) April 25th 2014.

## Background

Aging negatively affects structure and mechanical function of collagen-rich tissues [1–3]. The skin loses elasticity, blood vessels become stiffer, and tendons may become more compliant [4]. The possible age-related decline in tendon stiffness may in turn reduce maximal skeletal muscle performance [5] and postural balance [6], which negatively affects mobility in old age. Regular loading of tendons seems to protect the tissue from the negative effects of aging [7, 8] by increasing tensile stiffness [9–11]. However, the knowledge about training duration and the magnitude of load needed to induce favorable adaptations in tendons of older adults is limited. Moreover, the relation between tendon mechanical properties and molecular changes in the matrix is largely unresolved.

Training with heavy load seems to elicit a more pronounced increase in tendon stiffness than training with moderate loads in middle aged [12] as well as older adults [11], although low load training has also been demonstrated to affect tendon mechanical properties [4]. Short term (12 weeks) resistance training has effectively increased tendon stiffness in older adults in most [9–11] but not all [13] studies. Only few studies have investigated the effect of very long training duration and found no additional effect on tendon stiffness after either 18 months (older adults) [10] or 4 years (young adults) [14] compared to only three months of training. However, young athletes engaging in sports where one leg is more loaded than the other over several years display pronounced differences in tendon stiffness and cross-sectional area, suggesting a continued adaptation to loading beyond the first three months [15]. Thus, more studies are warranted to investigate the influence of training load over a long duration of training on tendon size and mechanical properties, especially in older adults.

The main contributor to tendon tensile stiffness is type I collagen, which makes up most of the tendon matrix. Acute exercise increases tendon collagen expression in animals [16] and peritendionus collagen synthesis in humans [17], but the overall collagen content and the collagen fibril volume fraction as measured by transmission electron microscopy (TEM) does not seem to be responsive to training [8, 18]. Moreover, recent evidence suggest that the vast majority of core tendon collagen molecules are not renewed after late teenage years [19], suggesting that the aging and loading induced adaptations in tendon mechanical properties are mediated by other molecular components in the matrix.

Another important matrix component that could influence tendon mechanical properties are collagen cross-links [20], which are essentially either enzymatic or non-enzymatic. Enzymatic cross-links (i.e. lysylpyrridinoline (LP) and hydroxylysylpyrridinoline (HP)) are tightly regulated in time and space by the family of lysyl oxidases (LOX) and are essential for normal tendon development. Tendon LOX expression is unaffected by regular training [21] but induced by acute exercise [16] indicating a stimulatory effect of exercise on formation of cross links. Life-long endurance training does not seem to affect HP or LP [8], but it remains unknown if long-term resistance training increases enzymatic cross-link density in older adults and if this is related to increased tendon mechanical properties.

Non-enzymatic cross-links form sporadically when reducing sugars react with amino-groups and mature into advanced glycation end-products (AGEs) [1, 2]. AGEs accumulate in collagen rich tissues with age [22], not least in tissues with slow turnover rate like tendon [23, 24], where they may eventually impair material properties [3, 25, 26]. Despite the slow turnover rate of tendon, regular training seems to reduce [21] or at least be associated with an attenuated AGE accumulation in older adults [8], and heavy resistance training reduces patellar tendon pentosidine, which is a cross-linking AGE, in young men [27]. However, it is still unknown how regular heavy and light load resistance training affects tendon AGEs in older adults. Moreover, although cross-linking AGEs increase tendon mechanical properties in animals [26, 28], they may also have the opposite effect by inducing fibril damage accumulation [3, 25] or by taking up space in the matrix [29], and the relation between tendon AGEs and mechanical properties in old humans needs to be clarified [30].

The present investigation aimed to compare the effects of long-term (12 months) heavy or light load resistance training on patellar tendon mechanical properties, morphology and collagen cross-links in older adults as well as the relation between changes in mechanical properties and tendon matrix. Our hypothesis was that compared to a control group, both training loads would increase patellar tendon stiffness. Moreover, we hypothesized that the increased tendon stiffness would primarily be related to greater density of enzymatic cross-links, whereas AGE cross-link accumulation would be attenuated and collagen content and fibril morphology would remain unchanged.

## Methods

### Study design

The present investigation was a sub-study of a randomized trial started in 2014 at the Institute of Sports Medicine Copenhagen [31]. The study primarily investigated the effect of 12 months resistance training on muscle strength and physical function in 450 men and women, 62–70 years old. The 36 participants recruited for the present sub-study gave consent to undergo additional tendon specific tests. They were allocated to 12 months of moderate load resistance training (MRT), heavy load resistance training (HRT), or habitual physical activity level (< 1 h strenuous exercise/week) (CON).

### Participants

Based on expected changes in tendon fluorescence of approximately 20%, which is what has been observed previously for pentosidine (a marker of AGEs) [27], and using the observed pooled SD of approximately 200 found in the present investigation, we needed 10 participants in each group to reach 80% power. The study included 17 women and 19 men, age 62–70 years. The participants were all home-dwelling, independent, and untrained. Exclusion criteria were more than 1 h of systematic strenuous exercise per week, systemic hormonal−/anti-hormonal therapy, anticoagulant therapy, a history of patellar tendinopathy within the past 12 months, chronic knee pain or other musculoskeletal problems impeding participation in resistance training. For the full list of exclusion criteria we refer to the original study protocol [31].

### Interventions

The interventions are described in detail in a previous publication [31]. Briefly, HRT was a supervised, whole body, progressive, heavy resistance training program. In the first 6–8 weeks of the intervention the participants performed 3 sets of 15 repetitions at a moderate load (~ 50–60% of one repetition maximum (1RM)) to accustom them to the exercises. After this, the intervention consisted of periods of 8 weeks in which the number of repetitions was gradually reduced from 12 to 6 while the load was accordingly increased from 70 to 85% of 1RM. The periods of 8 weeks were repeated throughout the 12 months intervention, and one week of restitution (no training) was interspersed between each period. The leg exercises targeting the patellar tendon were leg press and knee-extension performed in Technogym fitness machines (TechnoGym, Gambettola, Cesena, Italy).

MRT was a home-based, whole body, progressive, moderate load resistance training program performed as circuit training and using TheraBand® elastic rubber bands (Hygenic Corp., Akron, OH, USA) and the participants own body-weight. The number of repetitions was gradually increased from 3 sets of 12 to 3 sets of 18 repetitions. Load was increased whenever the participants could perform 3 sets of 18 repetitions without feeling exhausted, at which point the number of repetitions were once again reduced to 3 sets of 12. The exercises targeting the patellar tendon were squat and knee-extension. Training frequency was three times per week, and the participants were supervised once a week. Participants recorded training activity (load, number of sets and repetitions) in training diaries.

CON was encouraged not to change their habitual physical activity level, which was < 1 h of regular strenuous physical activity per week, and were offered participation in social/cultural activities approximately once a month.

### Muscle strength assessment

An investigator blinded to group allocation used a Good Strength dynamometer (V.3.14 Bluetooth; Metitur, Finland) to assess isometric knee extensor strength (IsoMVC) at 70^0^ knee flexion. The participants performed one familiarization trial before recording at least three verbally encouraged maximal isometric knee-extensions. Measurements were continued until no further improvement occurred [31].

### Muscle size

An experienced radiographer blinded to group allocation acquired MRI of the thighs using a 3.0 T TX Philips Achieva scanner (Philips Healthcare, Amsterdam, The Netherlands) with a 32-channel body array coil. The scanning was a 2D axial T1-weighted sequence (TR/TE = 666/20 ms, FA = 90, 672 × 672 matrix, 3 stacks with 3 slices, gap 1.91 mm, in-plane resolution 0.8 mm, slice thickness 4 mm) acquired at 10, 20 and 30 cm above tibia plateau, with three slices at each level for delineation of vastus lateralis of the quadriceps muscle and calculation of cross-sectional area. Image analysis was conducted by blinded assessors using the medical imaging software package Jim version 6.0 (Xynapse Systems, UK). The reported values for vastus lateralis cross-sectional area (VL-CSA) are from one measurement 20 cm proximal to the tibia plateau. In a few cases, the slice at the same location at follow-up was not visually consistent with that at the baseline measurement, due to small variations in the location of the slice matrix. Instead the most consistent neighboring slice (closer to 19.4 or 20.6 cm proximal to the tibia plateau) was used. We report VL-CSA of the dominant leg only [31].

### Blood measurements

A general health screening was performed at baseline including several analyses on blood samples. Blood samples were obtained by medical doctors or medical students. Blood analysis was performed with standard assays and kits at the clinical biochemistry department at Bispebjerg Hospital (Copenhagen, Denmark). We report here values for HbA1c and total cholesterol since they may affect tendon health and mechanics [32–35].

### Physical activity level

Physical activity level was measured with a previously validated [36] accelerometer and inclinometer (activPal micro, PAL technologies, Glasgow, Scotland), which was mounted on the thigh of the participants for five consecutive days, always including the weekend. Data were extracted with activPal software (Research edition, V.7.2.32, PAL Technologies, 2013). Here we report daily step count as a measure of physical activity level.

### Patellar tendon mechanical properties

The procedure for testing patellar tendon mechanical properties is a validated method, which has been used previously in our own and other laboratories [11, 13, 37]. The participants initially performed 5 min warm-up on a cycle ergometer (Monark, Sweden) at a low resistance to precondition the patellar tendon. The participants were then seated in a custom-made rigid chair with the hip- and knee-joints fixed in a 90^0^ angle. They then performed 8 s ramped isometric contractions with simultaneous recording of knee extensor force using a dynamometer (Noraxon Telemyo 2400 T G2, USA) and ultrasound (US) videos of the patellar tendon with a 10 MHz, 100 mm linear array transducer (Hitachi Hi Vision, Ascendus, Tokyo, Japan) to assess tendon elongation. We recorded 4–6 ramps to ensure at least 2–3 satisfactorily completed ramped contractions with a steadily increasing force production and ultrasound videos with a good contrast. US probe positioning at POST measurement was reproduced by comparison to the PRE measurement recordings.

Patellar tendon elongation was defined as the change in distance between the patella and tibia insertions of the tendon during the ramped contractions, and analyzed with a previously validated custom made semi-automated software [38]. Patella and tibia movements were always tracked within the area of patellar tendon insertion to reduce error due to rotation of tibia and patella during the isometric contraction. Patellar tendon force was calculated by multiplying dynamometer force with the external moment arm (distance from the lateral center of the leg cuff to the lateral collateral ligament) and dividing by the internal moment arm estimated as a percentage (6.5%) of femur length [39]. Femoral length was estimated as the distance from the most lateral point of trochanter major to the lateral collateral ligament. The measurement of external moment arm showed good reproducibility from PRE to POST with a coefficient of variation (CV) of 1,3%, indicating reproducible positioning of the leg-cuff. Second order polynomials were fitted to the force-deformation data points in Sigma Plot (Version 10.0, Systat Software, Germany) and used to estimate maximal values for tendon stiffness at the top 10% of the force-deformation relationship. Tendon deformation was normalized to strain by dividing with initial tendon length, and force was normalized to stress by dividing with average (total) tendon cross sectional area (CSA). Young’s modulus was calculated as the stress to strain ratio also in the top 10% of the stress-strain curve.

The investigators performing data analysis selected two trials for further analysis based on the following selection criteria: Visual consistency between bone movement and tracking points, good synchronization between force and deformation, a smooth inclining force curve, return to baseline after relaxation, and all other things equal, the trials with highest force and/or deformation were chosen. Because the force-deformation relationship of tendon is nonlinear, the raw data in the selected curves was cut off at the highest common force across repeated measurements for each individual.

US videos from 8 participants were analyzed using a custom implementation of cross-correlation tracking in Matlab (R2015b, MathWorks Inc., USA) due to technical difficulties with the video analysis in the program otherwise used. In all instances, we made sure to reanalyze both 0 and 12 months measurements so repeated measures on the same subject were analyzed with the same method.

### Patellar tendon dimensions

Patellar tendon length and CSA were assessed with a different MR-scanner than that used for muscle size determination. MRI is a reliable and accurate method for determining patellar tendon dimensions [40]. Trained radiographers performed the scans on a Signa Horizon LX 1.5 T MRI scanner (General Electic, Milwaukee, WI, USA) with an axial and sagittal T1-weighted turbo spin echo sequence (TE: 17; TR: 500; matrix: 512 × 512; FOV: 150 mm; Slice thickness: 3 mm), which has been used in previous human studies in our department [41]*.* The axial slices of the patellar tendon were positioned orthogonal to the length in the sagittal plane covering the distal patellar pole to the tibia insertion. A supportive pillow was placed in the knee coil to ensure slight stretch on the tendon by bending the knee, which made it easier to measure tendon dimensions. A phantom containing 1.0% CuSO_4_ was included in the image and subsequently used to adjust contrast settings.

All participants were scanned in their habitual state in the afternoon, and were instructed to avoid strenuous physical activity in the preceding 48 h to avoid the possible influence of training on tendon and muscle water content [42].

**Fig. 1:**
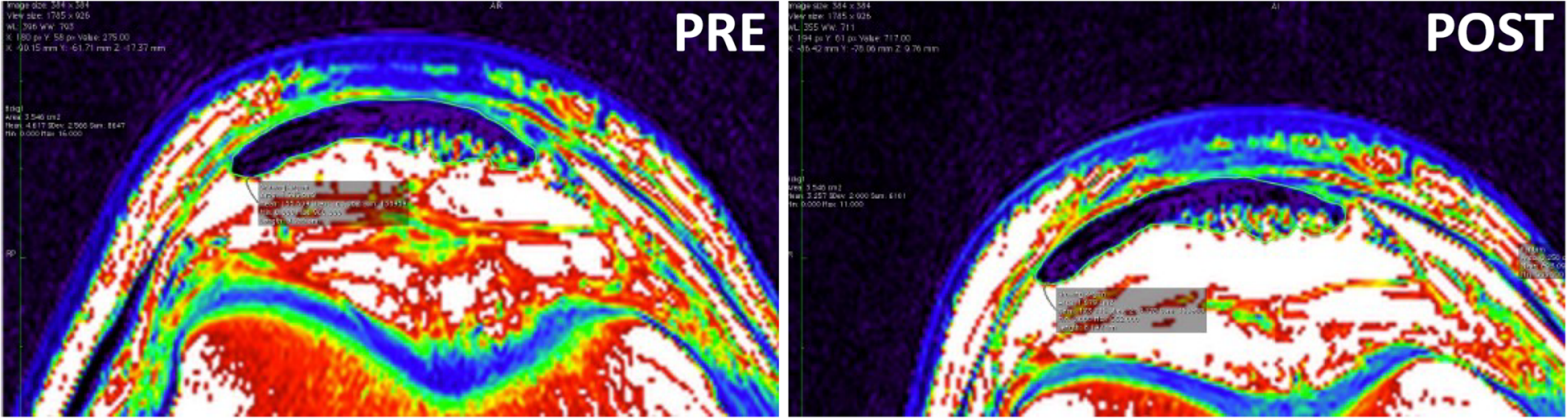
Typical magnetic resonance images of the proximal patellar tendon from the same subject before (PRE) and after (POST) 12 months intervention. The two images are obtained at the same location

Patellar tendon dimensions were assessed using Osirix imaging software (version 2.7.5, Osirix Imaging Medical, Geneva, Switzerland) to manually outline patellar tendon length as well as CSA at three locations (proximal, mid, distal) along the length of the tendon [15, 40]. All images were adjusted according to the phantom and measured using NIH (National Institute of Health) color scale, because this method provides more accurate measurements of patellar tendon CSA [40]. The proximal CSA was measured just distal to the patellar insertion*,* the distal CSA was measured just proximal to the tibia insertion, and the mid CSA on the slice midway between the proximal and distal slices. Using this procedure, tendon CSA was assessed at the same location at PRE and POST measurements (Fig. 1). Patellar tendon length was measured as the distance from the most dorsal insertion on the patella apex to the most dorsal insertion on the tibia. Coefficient of variation corrected for small sample size on the triplicate measurements was on average 1.5% (range: 0.2–3.2%) for patellar tendon length, and 2.3% (range: 0.2–6.1%) for total patellar tendon CSA. Day to day variation in measurements yielded a CV of 2.4%.

### Tendon biopsies

The patellar tendon biopsy procedure is a sterile procedure, which has been performed previously in our lab [43]. Briefly, the biopsy was obtained in local anesthesia (1 ml lidocaine, Lidokain Mylan 1 mg/ml, Mylan, Oslo, Norway) through a medio-lateral skin incision just distal to the patella with a semi-automated biopsy instrument (Bard Magnum, Bard biopsy systems, USA) at an angle of 45^0^ relative to the patellar tendon in the proximal to distal direction.

We obtained all biopsies at the same time of day before and after the intervention (± 1 h) to avoid the potential influence of circadian rhythm on tendon physiology [44] The non-dominant leg was biopsied before and the dominant leg after the intervention to avoid the influence of the first biopsy on the second [45]. The same trained physician obtained or supervised all biopsies except for four biopsies obtained by another experienced physician. Training was initiated between 5 and 42 days after the PRE biopsy with an average ± SD of 17 ± 9 days. Besides local soreness upon palpation, none of the participants reported any pain in the patellar tendon or other limitations when performing the prescribed exercises. Another investigator prepared the tissue for further analysis under light microscope. The tissue was kept moist in isotonic saline during the entire procedure. The biopsy was first dissected free from potential non-tendinous tissue (i.e. fat and subcutaneous tissue). Then, the investigator cut a small piece for electron microscopy with visible regular, longitudinal arrangement of the collagen fibers, which was immersed in glutaraldehyde and stored at 5 °C. The rest of the sample was immediately frozen in liquid nitrogen and subsequently stored at − 80 °C until further analysis.

### Fibril morphology

Fibril morphology was analyzed with transmission electron microscopy (TEM). Preparation of the samples are described in detail in previous publications [43, 46]. Images were obtained with a Philips TM 100 transmission electron microscope at 80 kV equipped with Megaview 2 camera. A blinded (to group) technician was instructed to zoom to a magnification of 1050 (100x100μm), where the tissue microstructure was not visible, and identify two different areas with core fibrillary structure (fascicles). The technician then divided the screen visually in six fields, and imaged one area in each field at 24500 x magnification (4x4μm). In this way we obtained a total of 12 unbiased images per biopsy, which all contained core tendon tissue with clearly visible cross-sectioned collagen fibrils.

One blinded investigator performed all measurements of fibril diameters in the image analysis software ImageJ (Version 1.51n FIJI package; NIH, Bethesda, Maryland, USA). A macro was initially applied on each image to randomly generate a 300 × 300 nm^2^ unbiased counting frame with guard regions, and semi-automatically delineate fibril contours by best fitting ellipses. The investigator then confirmed the automated measurements within the counting frame, and manually corrected any erroneously measured fibrils. We used an elliptic fit because some fibrils were not perfectly round, and chose the minor axis diameter as the true fibril diameter to alleviate the influence of sectioning angle. The investigator counted on average 335 ± 131 fibrils for each tendon specimen (12 images). The data were reduced for each individual and time point to mean fibril diameter, volume fraction, and fibril density.

### Biochemical analysis

The raw tendon samples were thawed and freeze-dried to give the tendon dry-weight. For biochemical analysis, we used on average 7.2 mg (1.9 to 17.8 mg) tissue with an average water content of 84.2%, giving 1.1 mg tissue dry weight for biochemical analysis. Afterwards, we performed gas-phase hydrolysis on the samples within sealed and evacuated tubes for 24 h at 110 °C followed by freeze-drying the samples again. The freeze-dried samples were dissolved in de-mineralized water to a concentration of 5 mg/ml.

Hydroxy-proline was determined on the hydrolyzed samples as a measure of collagen content as described in detail in a recent publication [47].

HP and LP were determined with two different ELISA kits, one made for measuring HP in serum (MicroVue Serum PYD, 8019, Quidel Corp.), and one for measuring LP in urine (MicroVue DPD, 8007, Quidel Corp.). Due to the much higher concentration of HP and LP in the tissue hydrolysates than in serum and urine, the samples (reconstituted in water at 5 mg/mL) were diluted 2000 fold for the HP analysis and 100 fold for LP, which reduces the risk of interference from compounds that would not normally be present in serum or urine. Both assays were competitive ELISAs based on a polyclonal rabbit antibody for HP and a monoclonal one for LP. The ELISAs were performed in duplicate with a CV of 6 and 14% for LP and HP, respectively. Aside from using a different type of sample the manufacturer’s instructions were followed without modification.

Total fluorescence was used as a marker of total AGE modification [48]. Fluorescence measurements were made on the hydrolysates reconstituted in water. To ensure a consistent pH and get enough volume for triplicate measurements, samples were diluted 6 fold into 0.12 M HCl (0.1 M final concentration). The diluted samples containing 83 μg tissue/ml were plated onto black 96 well plates and read on a Wallac1420 Victor microplate reader (Perkin Elmer) at 340/5 nm excitation and 460 nm (382–507 nm) emission. Since fluorescence correlated positively to collagen content (r^2^ = 0.52, *p* < 0.001), we chose to report fluorescence relative to collagen.

### Statistics

Data were analyzed by repeated measures two-way ANOVA with baseline adjustment using time-point (0 mths vs 12 mths) and intervention-group (CON vs MRT vs HRT) as factors and Tukey-Kramer post-hoc tests. Normality was confirmed by visual inspection of residual plots. Signal intensity and fluorescence were not normally distributed and were log-transformed and analyzed again. All other variables were normally distributed. Outlier analysis was performed with an online Grubb’s test [49] before statistical testing (no outliers were detected). SAS statistical Software v. 9.4 (SAS Institute, USA) were used for statistical testing. Continuous variables are presented as arithmetic mean ± SE, and log-transformed values are presented as geometric mean [upper limit-lower limit]. Participant characteristics were summarized as mean ± SD.

## Results

### Participants

One woman dropped out of CON due to lack of time, and one woman and one man dropped out of MRT due to stroke and meningitis. The remaining 33 participants completed the study with an average training compliance of 86 ± 12%, which was not different between MRT and HRT. Their physiological characteristics are presented in Table 1.

**Table 1:** Participant baseline characteristics

	Total	CON	MRT	HRT
Participants	33	10	13	10
Sex (men/women)	18/15	6/4	5/8	7/3
Age (y)	67 ± 2	68 ± 1.8	66 ± 2.4	67 ± 2.3
Height (cm)	173 ± 8	175 ± 8	173 ± 7	171 ± 8
Weight (kg)	78 ± 14	82 ± 16.9	73 ± 12	79 ± 14
BMI (kg/m^2^)	25.8 ± 3.7	26.6 ± 4.3	24.3 ± 3.5	26.9 ± 3.1
HbA1c (mmol/l)	36 ± 3.1	37 ± 3.5	35.2 ± 3.0	35.4 ± 2.9
Total-C (mmol/l)	5.9 ± 0.9	5.7 ± 0.8	6.0 ± 1.0	6.0 ± 0.9
Training Compliance (%)	86 ± 12		86 ± 16	86 ± 7

Values are mean ± SD. Baseline group-differences within studies evaluated with one-way ANOVA, chi-square test (sex distribution), and Welch’s unpaired t-test (compliance). No significant group differences

*HRT* heavy resistance training, *MRT* moderate load resistance training, *CON* control, *BMI* body mass index, *Total-C* total cholesterol

### Muscle strength and size

**Fig. 2:**
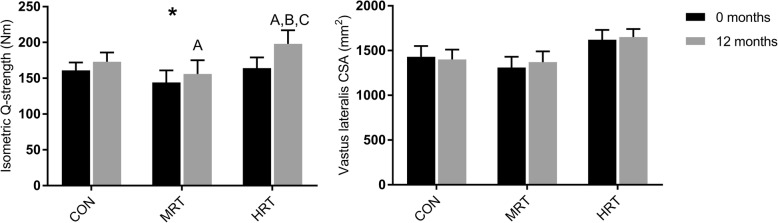
Isometric quadriceps (Q)-strength and vastus lateralis cross-sectional area (CSA) before (0 months) and after 12 months heavy resistance training (HRT), moderate load resistance training (MRT) or no training (CON). *Significant time x group interaction based on 2-way repeated measures ANOVA (*p* < 0.05) **a**: Significantly different from 0 months (*p* < 0.05). **b**: Significantly different from CON12 (*p* < 0.05), and **c**: Significantly different from MRT12 (*p* < 0.05) based on Tukey-Kramer post-hoc test

Twelve months intervention significantly improved isometric quadriceps muscle strength in HRT (*p* < 0.01) and MRT (*p* < 0.05) but not in CON. There was a significant time x group interaction since HRT improved more than both CON and MRT (p < 0.05) (Fig. 2). VL-CSA was not significantly affected by training (Fig. 2).

### Health related variables & physical activity level

Weight, BMI, total cholesterol and HbA1c displayed no time x group interactions. There was a significant main effect of time (*p* < 0.001) on HbA1c which was higher after the intervention in all three groups. All other health-related variables did not change significantly during the intervention. Physical activity level measured as daily step-count displayed no time x group interactions, but there was a trend towards a main effect of time with increasing step-count across groups from 8500 (0 months) to 9400 (12 months) (*p* = 0.07).

### Patellar tendon mechanical properties

**Fig. 3:**
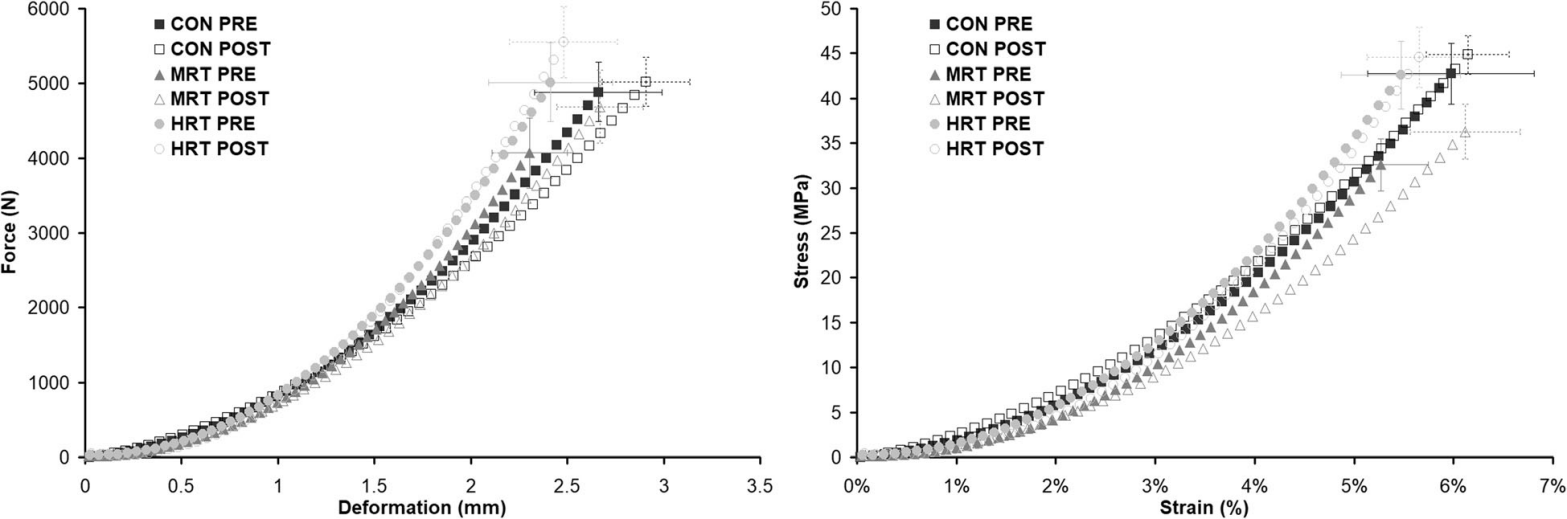
Illustration of average patellar tendon force/deformation (left) and stress/strain (right) curves before (PRE) and after (POST) 12 months heavy load resistance training (HRT), moderate load resistance training (MRT), or no training (CON)

Average patellar tendon force/deformation and stress/strain curves are illustrated in Fig. 3. There were significant time x group interactions in maximal patellar tendon stiffness and Young’s modulus. Post hoc tests showed higher maximal stiffness and a tendency for higher maximal modulus (*p* = 0.09) with HRT compared to CON (Table 2), and maximal stiffness tended to be higher after HRT compared to MRT (*p* = 0.06). Maximal deformation, strain, force, and stress did not display any time x group interactions, but there was a significant main effect of time in all these variables, except for maximal stress (*p* = 0.07).

**Table 2:** Maximal patellar tendon mechanical properties

	CON (*n* = 10)		MRT (*n* = 13)		HRT (*n* = 10)	
	0 mths	12 mths	0 mths	12 mths	0 mths	12 mths
Internal moment arm (cm)	2.75 ± 0.04		2.79 ± 0.04		2.73 ± 0.05	
Maximal deformation (mm) ^**‡**^	2.7 ± 0.3	2.9 ± 0.2	2.3 ± 0.2	2.7 ± 0.2	2.4 ± 0.3	2.5 ± 0.3
Maximal force (N) ^**‡‡**^	4890 ± 390	5020 ± 330	4070 ± 480	4690 ± 490	5020 ± 530	5550 ± 470
Maximal stiffness (N/mm)^**^	3530 ± 490	3010 ± 440	3350 ± 300	3170 ± 210	4060 ± 430	4420 ± 340^B^
Maximal strain (%) ^**‡**^	6.0 ± 0.8	6.4 ± 0.5	5.3 ± 0.5	6.1 ± 0.6	5.5 ± 0.6	5.7 ± 0.5
Maximal stress (MPa)	43 ± 3	43 ± 2	33 ± 3	36 ± 3	43 ± 4	45 ± 3
Maximal modulus(MPa)^*^	1430 ± 2000	1200 ± 180	1180 ± 70	1090 ± 50	1510 ± 150	1560 ± 140

Values are means ±SE. Data analyzed by repeated measures two-way ANOVA with baseline adjustment and Tukey-Kramer post hoc tests

*CON* control, *MRT* moderate load resistance training, *HRT* heavy resistance training

Significant interaction denoted by *(p < 0.05) or **(p < 0.01). Main effect of time denoted by ‡(p < 0.05) or ‡‡(p < 0.01). Post hoc tests: B: significant difference from CON12 (p < 0.05)

**Fig. 4:**
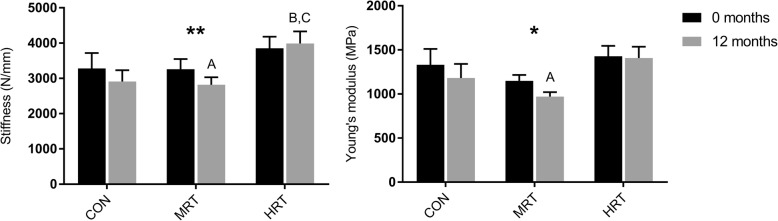
Common force patellar tendon (PT) stiffness and Young’s modulus before (0 months) and after 12 months heavy resistance training (HRT), moderate load resistance training (MRT) or no training (CON). Bars represent mean ± SE. Significant time x group interaction denoted by **(*p* < 0.01) or *(*p* = 0.05) based on repeated measures 2-way ANOVA. A: Significantly different from 0 months (p < 0.05). B: Significantly different from CON12 (p < 0.05), C: Significantly different from MRT12 (p < 0.05) based on Tukey-Kramer post-hoc test

Common force mechanical properties also displayed a significant time x group interaction in patellar tendon stiffness and a strong trend towards interaction in Young’s modulus (*p* = 0.05) (Fig. 4). Common force stiffness was significantly higher after HRT compared to both CON and MRT, which reduced this variable. Common force Young’s modulus further tended to be higher after HRT compared to MRT (p = 0.07), and MRT significantly reduced modulus (Fig. 4). Common force deformation and strain displayed no time x group interactions or main effects of time.

### Patellar tendon MRI

**Fig. 5:**
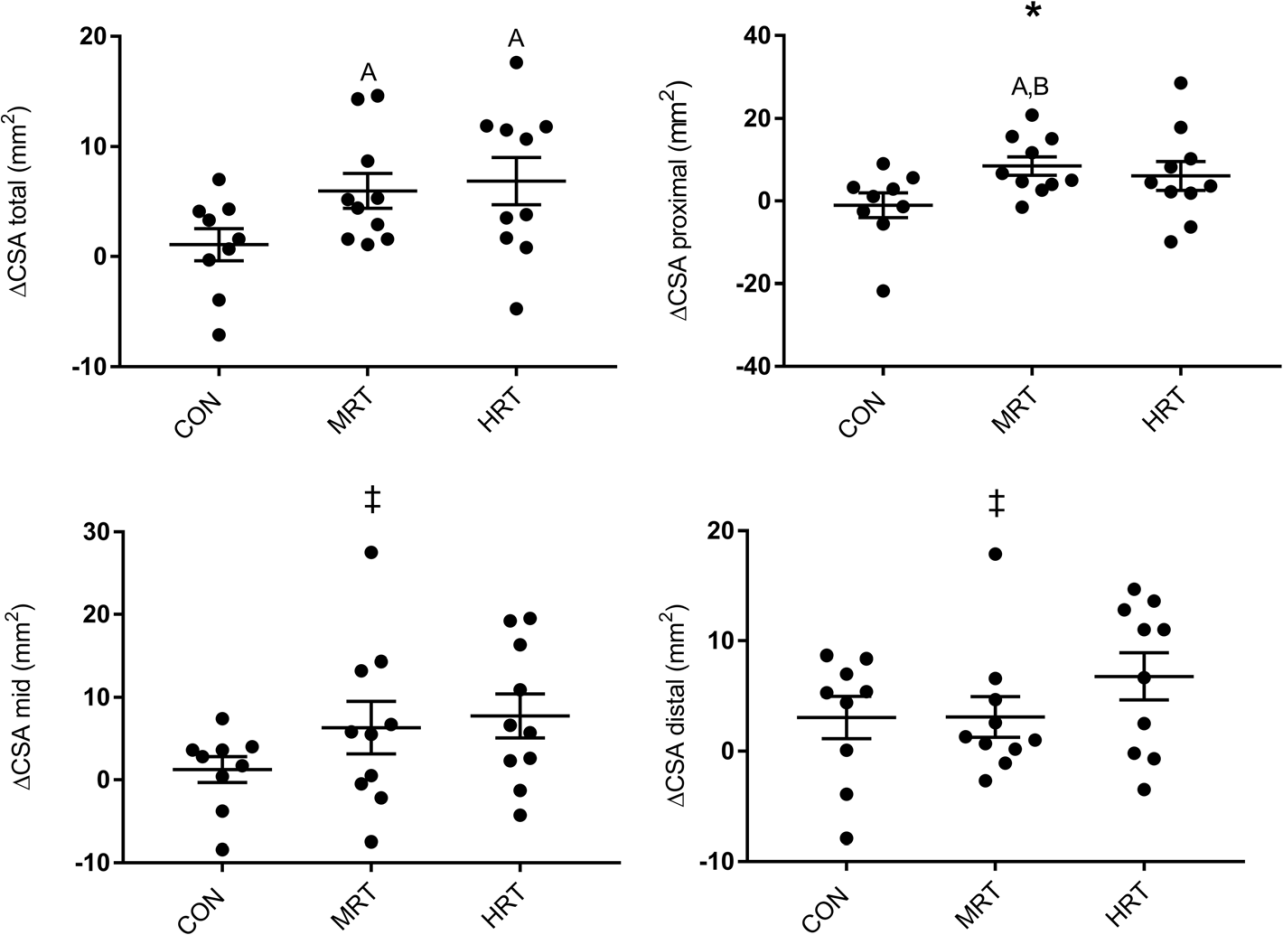
Changes in patellar tendon cross-sectional area (CSA) in three different regions (proximal, mid, distal) and in total after 12 months heavy resistance training (HRT), moderate load resistance training (MRT) or no training (CON). Bars represent mean ± SE. *Significant time x group interaction based on repeated measures ANOVA (pz0.05). ‡: Main effect of time (*p* < 0.05). **a**: Significant change from 0 to 12 months (*p* < 0.05), **b**: Significantly different from CON (*p* < 0.05), **c**: Significantly different from MRT (*p* < 0.05) based on Tukey-Kramer post-hoc test

There was a trend towards a time x group interaction of total patellar tendon CSA (p = 0.07), which increased significantly over time in HRT and MRT but not in CON (Fig. 5). The proximal tendon region (CSA prox) showed a significant time x group interaction, and post-hoc test revealed increase over time in MRT and a similar, although not significant, increase in HRT (*p* = 0.25) (Fig. 5). The mid and distal tendon regions did not display any time x group interactions, but there was a main effect of time in both regions where CSA increased from 0 to 12 months across groups.

### Collagen fibril morphology

There were no significant time x group interactions in volume fraction, fibril diameter or fibril density (Table 3). There was a main effect of time in fibril diameter, which decreased over time, and fibril density which increased over time.

**Table 3:** Collagen fibril morphology

	CON		MRT		HRT	
	0 mths (*n* = 9)^a^	12 mths (*n* = 9)^a^	0 mths (*n* = 12)^a^	12 mths (*n* = 12)^a^	0 mths (*n* = 9)^a^	12 mths (*n* = 9)^a^
Volume Fraction (%)	63 ± 2	59 ± 1	59 ± 2	62 ± 1	59 ± 2	59 ± 2
Mean fibril diameter (nm)^‡^	91 ± 5	83 ± 4	87 ± 3	86 ± 5	89 ± 4	78 ± 3
Density (#/μm^2^)^‡^	27 ± 2	31 ± 3	28 ± 2	33 ± 3	28 ± 3	36 ± 3

Values are means ±SE. Data analyzed by repeated measures 2-way ANOVA with baseline adjustment and Tukey-Kramer post-hoc test

*HRT* heavy resistance training, *MRT* moderate load resistance training, *CON* no training

Main effect of time denoted by ‡(p < 0.05)

^a^One value missing due to technical problems

### Collagen and collagen x-links

**Fig. 6:**
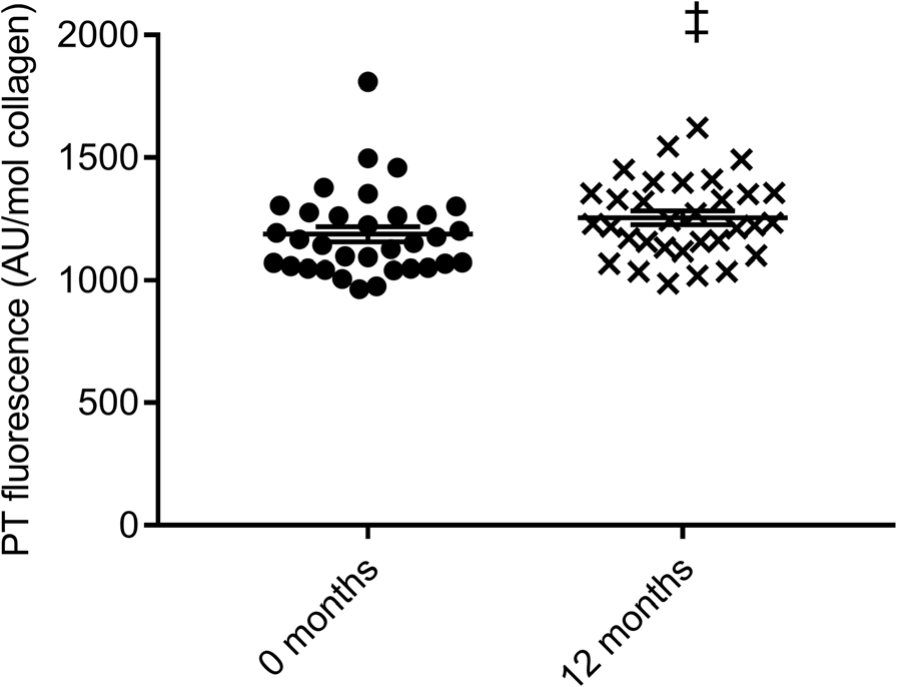
Fluorescence of patellar tendon (PT) biopsies as a marker of non-enzymatic (AGE) cross-links before (0 months) and after 12 months intervention. ‡: Main effect of time (*p* < 0.05) based on repeated measures two-way ANOVA with baseline adjustment. AU = arbitrary units

There were no significant time x group interactions in tendon collagen content, enzymatic cross-links, or fluorescence (Table 4). Tendon fluorescence displayed a significant main effect of time (Fig. 6).

**Table 4:** Collagen and collagen cross-links

	CON		MRT		HRT	
	0 mths (n = 10)	12 mths (n = 10)	0 mths (n = 13)	12 mths (n = 13)	0 mths (*n* = 9)^a^	12 mths (n = 9)^a^
Collagen (%)	69 ± 3	62 ± 2	61 ± 4	59 ± 2	63 ± 3	61 ± 2
HP (mmol/mol collagen)^b^	298 ± 28	322 ± 25	314 ± 23	267 ± 21	254 ± 28	292 ± 22
LP (mmol/mol collagen)^b^	24.8 ± 5.0	26.5 ± 3.8	40.8 ± 4.4	28.8 ± 5.1	37.0 ± 5.9	34.3 ± 3.2
Fluorescence (AU/mol collagen)^‡^	1139 [1096–1185]	1235 [1176–1297]	1155 [1124–1187]	1196 [1161–1232]	1263 [1191–1339]	1328 [1285–1372]

Values are means ±SE or geometric mean [lower limit – upper limit]. Data analyzed by repeated measures two-way ANOVA with baseline adjustment and Tukey-Kramer post hoc test

CON = Control, MRT = moderate load resistance training, HRT = Heavy load resistance training

No time x group interactions. ‡: Main effect of time (p < 0.05)

^a^One missing sample due to anti-coagulant therapy. ^b^Five missing pairs of samples due to logistics

CON: n = 9, MRT: *n* = 11, HRT: *n* = 8

## Discussion

The present investigation compared the effects of high or moderate load long-term resistance training on patellar tendon mechanical properties, macro- and microscopic morphology, and collagen cross-links in older adults. The main findings were that adaptation of patellar tendon mechanical properties over the 12 months intervention was dependent on magnitude of training load, whereas tendon CSA increased after both heavy and moderate load resistance training. Contrary to our hypothesis, the load-dependent changes in tendon mechanical properties were unrelated to any changes of enzymatic cross-links or advanced glycation end-products.

Strength training improved maximal isometric quadriceps strength (IsoMVC) with heavy load training (+ 21%) being superior to moderate load (+ 8%) or no training (7%) (Fig. 2). The superior effect of heavy compared to moderate load training on muscle strength is consistent with current knowledge [50] although the strength increase in the present study was slightly lower than previously reported in a meta-analysis of strength training studies [50]. Vastus lateralis CSA was surprisingly not affected by training, suggesting that either there was a quite large neural adaptation to training or that muscle hypertrophy mainly occurred in the other components of the quadriceps muscle. The relatively high training compliance of 86% in both groups could not explain the blunted muscular response to training. The training load and volume in the present investigation was comparable to previous investigations [50], but we cannot exclude that the prescribed training load and volume was compromised for some participants due to discomfort or lack of motivation. Another possibility is that the population of older adults in the present study had a higher fitness level to begin with than in previous investigations, which is supported by a relatively high habitual physical activity level of ∼8500 daily step-counts.

### Patellar tendon mechanical properties

Tendon stiffness calculated at common force increased by 6% after HRT, and decreased by 10 and 7.5% after MRT and CON respectively. Young’s modulus remained unchanged after HRT but decreased by 14% after MRT and 9% after CON. The strong trend towards different tendon responses in the three groups (*p* = 0.05) may suggest an age-dependent decrease of tendon mechanical and material properties, which was ameliorated by HRT but not MRT (Fig. 4). The fact, that tendon modulus and not only tendon stiffness was affected by training suggest that there was a change in material quality and not only amount of tissue. Although reduced stiffness and modulus over time in the CON group may be plausible, unchanged stiffness after 12 months heavy load training combined with relatively small differences between the three groups is somewhat surprising since previous studies have reported ∼20–60% increases of patellar tendon modulus after only three months heavy load training in older adults [9, 11, 13, 51]. The modest tendon adaptation however fits well with the modest muscular adaptation to training, since we would expect the muscle-tendon unit to adapt synchronously [52]. Although HRT did not increase tendon modulus, our findings support previous studies that have found superior effects of heavy compared to light load training on tendon mechanical properties in young and middle aged [12, 53] as well as older adults [11]. The novelty of the present finding is that moderate load training with an approximately comparable volume as heavy load training was also insufficient to affect tendon mechanical properties. Our results thus confirm that a certain loading threshold needs to be surpassed in order for tendons to adapt [54, 55]. In fact, MRT seemed to reduce tendon stiffness, which contradicted our own hypothesis as well as a previous study showing decreased strain of the vastus lateralis aponeurosis after low load training [4]. As for the CON group, MRT may have provided an insufficient mechanical stimulus on the patellar tendon to counteract an age-related decrease of stiffness over the 12 months intervention.

Several previous studies have investigated the effects of short-term (3 months) resistance training on tendon mechanical properties and found either increased [9–11, 51] or unchanged [13] tendon stiffness. Only few studies have investigated the effect of long-term training and found no additional effects after 1.5 [10] or 4 years [14] compared to three months training. We cannot exclude that the improvements in tendon stiffness and modulus measured after 12 months training were already present after the first three months. In another recent randomized training study in our department (unpublished data) including older adults with an average age similar to the ones in the present study, and using the same test-protocol, we found no significant differences in tendon mechanical properties after 3 months heavy or light load resistance training. Although many previous studies have found significant increases of patellar tendon stiffness and modulus after short-term resistance training in older adults [9, 11, 51], our own data suggest that longer training periods are superior. A more protracted mechanical adaptation to training would also fit with the relatively slow turnover of tendon extracellular matrix.

Taken together, our results show a more blunted response of the muscle-tendon unit to training compared to previous studies but confirm that heavy load training is superior with regards to inducing favorable mechanical and material adaptations of the patellar tendon. Maintenance of tendon stiffness may be critical to optimal muscle function [5, 56] and postural balance [6] in old age, and our results therefore support that heavy load resistance training should be incorporated in training programs in this age group.

### Patellar tendon morphology

Patellar tendon CSA (total) tended to be affected by intervention group (*p* = 0.07) with increases after both HRT (+ 6%) and MRT (+ 5%), but not CON (+ 1%) (Fig. 5). The possible increase in CSA makes the tendon able to support higher loads without imposing more stress on the tendon, and thus protects the tissue from damage. To our surprise, the effect of training on tendon CSA was not dependent on load magnitude since MRT in contrast to mechanical properties increased tendon CSA equally much as HRT (Fig. 5). The tendon tended to grow without any muscle hypertrophy, although myofibrillar protein synthesis is more sensitive to loading than tendon collagen synthesis [57]. Although somewhat surprising after a resistance training intervention, tendon growth is possible without a hypertrophic muscle stimulus as observed after life-long endurance running [8]. Since we only measured vastus lateralis CSA in the present investigation it cannot be ruled out that hypertrophy occurred in the remaining three muscle compartments of the quadriceps.

Although several short-term training studies have not been able to show any effect of heavy or light load on tendon CSA in older adults [9, 11, 51, 58], our longitudinal data supports some [8, 15], albeit not all [14] previous human cross-sectional data, which show that long-term (several years) habitual loading of both relatively low [8] and high load [15] is associated with higher patellar tendon CSA. One recent longitudinal study did, however, not find additional effect of 1.5 years compared to three months heavy resistance training on Achilles tendon CSA [10], and it is possible that specific tendons have unique time-courses of adaptation to loading. The increments of patellar tendon CSA tended to be region-specific with significant training effects in the proximal region, but not in the mid and distal regions (Fig. 5). A previous study in young men found similar region-specific increments of the patellar tendon CSA with 7% higher proximal CSA after light load training and 6 and 4% higher proximal and distal CSA respectively after high load training [59]. The increased CSA in that study was accompanied by increased tendon stiffness in the heavy load group only, which corroborates our own data. Local stress concentration in the patellar tendon during loading may explain the regional adaptations. Similar regional adaptations following strength training do not seem to take place in the Achilles tendon in older adults [10].

Increased CSA with training may be mediated by addition of new collagen to existing fibrils, new fibrils, increased intrafibrillary spacing between collagen molecules, or more interfibrillary material such as fat, water, or proteoglycans. In the present investigation we measured core tendon collagen content, fibril volume fraction, as well as fibril diameter and density but none of these variables were affected by training (Table 3). The lack of training effect on collagen content and fibril morphology was not surprising, since it has recently been demonstrated convincingly that no or very little renewal of collagen takes place in the core tendon after teenage years [19]. Moreover, it is also consistent with previous human cross-sectional data on master athletes compared to sedentary age-matched controls [8]. However, it is possible that adaptations at the fibrillary level have gone undetected in our core tendon biopsies, since new collagen may have been added to the peripheral region of the tendon [17] or to the most proximal region, which would be consistent with the region-specific adaptations in tendon CSA. Region-specificity could thus explain the disparity between adaptations at the fibrillary and whole tendon level. Training induced accumulation of water could also increase tendon CSA, but this explanation seems unlikely since water accumulation into the matrix would theoretically reduce fibril volume fraction which was unaffected by training.

In contrast to the changes in mechanical properties, we found that region specific patellar tendon hypertrophy occured independent of load magnitude. This could however not be explained by changes in the ultrastructural morphology of the tendon core which was unaffected by training.

### Collagen cross-links

Contrary to our hypothesis, the enzymatic cross-links HP and LP were unaffected by 12 months resistance training (Table 4). Previous human data have also shown no difference in enzymatic cross-links between endurance trained and sedentary older adults [8], and no effect of short-term resistance training in young adults [60]. Our measurements of LP in tendon were similar in magnitude to that found by others [20, 27, 30], but it should be acknowledged that our HP results were somewhat lower than that found in other studies [20, 27, 30, 60]. It cannot be excluded that our measurements may have underestimated the HP content, but importantly there was no change in determined HP after exercise. Our results thus confirm that enzymatic cross-links do not mediate moderate or high load training induced changes in tendon stiffness or Young’s modulus despite long training duration. Since enzymatic cross-links are essential to development of normal force transmission within the tendon [3, 20, 61], we also analyzed the relationship between tendon stiffness or modulus and HP or LP, at baseline as well as in the changes over time. The analysis showed no correlations between enzymatic cross-links and tendon mechanical properties in our group of older adults, which corroborate previous human and animal studies [30, 62, 63] and suggests that enzymatic cross-links play a limited role in the adaptation of tendon biomechanics after maturity. It is also plausible that enzymatic cross-links after maturity are only formed in the peripheral regions of the tendon, where there is also evidence of collagen synthesis in response to exercise [17], and future research should investigate region-specific molecular adaptations to training.

Fluorescence which was used as a marker of AGE content increased by ~ 6% over the 12 months across intervention-groups (Fig. 6), but was unaffected by both moderate and heavy load resistance training. The time dependent increase of fluorescence was not surprising given the nature of the glycation process, which results in accumulation of AGEs over the life-course [1]. It was however somewhat surprising that 12 months training could not attenuate AGE accumulation since others have found decreased tendon AGE-levels after short-term endurance training in old mice [21], attenuated accumulation of the AGE-marker pentosidine after life-long endurance training [8], and reduced pentosidine after short-term heavy load resistance training in young men with patellar tendinopathy [27]. Even longer training duration may be necessary for healthy old humans to significantly attenuate AGE-accumulation.

**Fig. 7:**
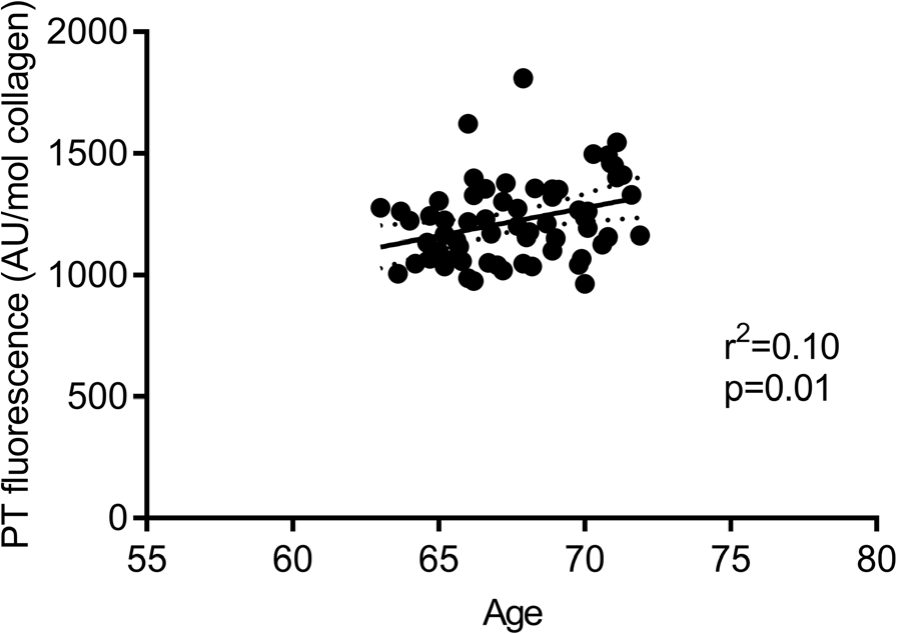
Correlation between age and patellar tendon (PT) fluorescence using bot PRE and POST values as determined by Pearson’s correlation coefficient. Dotted lines are 95% confidence bands of the best fit

To support that fluorescence measurement provided a truthful depiction of AGE-accumulation, we performed correlational analysis between age and fluorescence (Fig. 7). As expected, fluorescence correlated positively to age even within the relatively small age-range in our population (63–71 years). Interestingly, HbA1c also increased by ~ 3% or ~ 1 mmol/mol (*p* < 0.0001) without any difference between groups (Table 1). Increased HbA1c confirms the time-dependent accumulation of glycation products, but it may also be due to variation between PRE and POST measurements since the increase was way higher than what other large-scale studies on healthy individuals have found (~ 1 mmol/mol over 10 years) [64]. There was no relationship between tendon fluorescence and HbA1c either at baseline or in the changes over time, indicating different time-patterns of erythrocyte and tendon glycation.

We further examined the relationship between fluorescence and Young’s modulus since accumulation of AGEs may affect tendon material properties [3, 65], but there was no correlation between these variables either at baseline or in the changes over time. Previous in vitro studies have shown associations between AGEs and tendon mechanical properties [21, 26, 28], but human studies have in line with our results not been able to associate tendon AGEs and in vivo patellar tendon mechanical properties [30, 62], suggesting that other matrix components (i.e. proteoglycans, glycoproteins) contribute more to in vivo mechanical phenotype in old humans. AGEs comprise a large and heterogeneous family of chemical compounds, and it is possible that training induced changes of specific cross-linking AGEs have “drowned” in the measurement of total fluorescence. Further, region-specific changes in AGEs may have occurred in the outer layers or most proximal and distal tendon regions, without being detected in our core tendon samples. Finally, it is possible that AGEs mainly affect tissue mechanics in the failure region [47, 66, 67], whereas in vivo tendon testing only considers mechanical properties in the physiological range of the force-elongation relationship. It must be noted that recent studies have shown that the deformation mechanism and strength of the tendon are greatly affected by the presence of cross-links [68], and that multiscale mechanical analysis of in vitro glycated tendons strongly suggests that AGEs reduce tissue viscoelasticity by severely limiting fiber–fiber and fibril–fibril sliding [69].

Tendon fluorescence increased as expected over time and this may very well be due to age-related accumulation of AGEs. To prevent tendon AGE accumulation, training should be of longer duration than 12 months or probably initiated at younger age. The relation between patellar tendon AGEs and in vivo mechanical properties remains elusive.

### Limitations

In contrast to young women [70, 71], older postmenopausal women seem to have indistinguishable tendon mechanical properties from men [72]. Both men and women were included in the present study to make the results more generally applicable. Although not statistically significant, more men were randomized to HRT and more women were randomized to MRT. To test the potential influence of sex on the training effect, we performed a secondary 2-way ANOVA on the changes over time with sex and intervention-group as factors. The analysis did not show any interactions between sex and intervention-group or main effects of sex in muscle or tendon variables, suggesting that older men and women respond similarly to resistance training. This makes it unlikely that the unequal sex-distribution explained the group-differences in training adaptations.

Despite a high average training compliance of 86% in both MRT and HRT, the range was quite large (51 to 100%). We therefore made a secondary per protocol statistical analysis excluding participants with a compliance < 80% and also excluding one CON who admitted to have initiated strenuous training activities during the intervention. The remaining 7 HRT (compliance = 88 ± 5), 9 MRT (compliance = 94 ± 5), and 9 CON did not display appreciably different results than the primary statistical analysis and did consequently not affect the reached conclusions.

The duration of the ramped contractions was fixed at 8 s in the present investigation, which means that individuals with increased maximal muscle strength had a higher rate of force development. Higher rate of force development has been associated with increased tendon stiffness but this has mainly been shown at even higher rates of force development (i.e. 50 Nm/s (~ 3 s.) to 110 Nm/s (~ 1.5 s) [73] than in the present investigation. We therefore find it less likely that the group differences in maximal strength could explain the observed group differences in stiffness after the interventions.

Antagonist (hamstring) co-contraction may amount to 10–30% of the resultant knee-joint moment during isometric knee-extensions as measured by electromyography (EMG) [74]. In the present investigation we however chose not to consider this potential contribution to the knee-joint moment because it would add an additional level of technical variation to the estimation of tendon force, which in our experience (unpublished data) is even larger in untrained individuals due to EMG cross-talk (overestimated co-contraction due to quadriceps signal).

The present investigation was a nested study where the training interventions and study population were originally determined to investigate the impact of different training regimens on muscle function and general health in a representative elderly population. Although the study design may not have been ideally suited to answer the question about the specific influence of training load on tendons (i.e. lack of training volume matching, heterogeneity of the participants), our results still support that a training program designed to generally enhance muscle function and health in an elderly population also affected connective tissue function.

## Conclusion

The present study demonstrated load-dependent adaptations of patellar tendon mechanical properties with heavy load training being superior to moderate load or no training. However, patellar tendon cross-sectional area increased after training independent of load magnitude. The observed adaptations to resistance training could not be related to any changes in collagen content, fibril morphology, enzymatic cross-links, or advanced glycation end-products in the tendon core. We suggest that heavy load resistance training over longer periods is advisable for older adults to maintain tendon function with aging.

## Abbreviations

1RM: One repetition maximum
AGE(s): Advanced glycation end-product(s)
ANOVA: Analysis of variance
BMI: Body mass index
CON: Control group
CSA: Cross sectional area
CV: Coefficient of variation
EMG: Electromyography
HP: Hydroxylysylpyrridinoline
HRT: Heavy load resistance training
IsoMVC: Maximal voluntary isometric knee-extensor strength
LOX: Lysyl oxidase
LP: Lysylpyrridinoline
MRI: Magnetic resonance imaging
MRT: Moderate load resistance training
NIH: National Institute of Health
POST: After intervention (12 months)
PRE: Before intervention (0 months)
TEM: Transmission Electron Microscopy
Total-C: Total serum cholesterol
US: Ultrasound
VL-CSA: vastus lateralis cross-sectional area
